# Rotenone aggravates PD-like pathology in A53T mutant human α-synuclein transgenic mice in an age-dependent manner

**DOI:** 10.3389/fnagi.2022.842380

**Published:** 2022-08-08

**Authors:** An-Di Chen, Jia-Xin Cao, Hai-Chao Chen, Hong-Li Du, Xiao-Xia Xi, Jing Sun, Jie Yin, Yu-Hong Jing, Li-Ping Gao

**Affiliations:** ^1^Institute of Anatomy and Histology & Embryology, Neuroscience, School of Basic Medical Sciences, Lanzhou University, Lanzhou, China; ^2^Institute of Biochemistry and Molecular Biology, School of Basic Medical Sciences, Lanzhou University, Lanzhou, China; ^3^Center of Experimental Animal, School of Basic Medical Sciences, Lanzhou University, Lanzhou, China; ^4^Key Laboratory of Preclinical Study for New Drugs of Gansu Province, School of Basic Medical Sciences, Lanzhou University, Lanzhou, China

**Keywords:** Parkinson’s disease, human α-syn^+/–^ (A53T), rotenone, aging, brain–gut axis

## Abstract

Multiple factors such as genes, environment, and age are involved in developing Parkinson’s disease (PD) pathology. However, how various factors interact to cause PD remains unclear. Here, 3-month and 9-month-old hα-syn^+⁣/−^ mice were treated with low-dose rotenone for 2 months to explore the mechanisms that underline the environment–gene–age interaction in the occurrence of PD. We have examined the behavior of mice and the PD-like pathologies of the brain and gut. The present results showed that impairments of the motor function and olfactory function were more serious in old hα-syn^+/–^ mice with rotenone than that in young mice. The dopaminergic neuron loss in the SNc is more in old hα-syn^+/–^ mice with rotenone than in young mice. Expression of hα-syn^+/–^ is increased in the SNc of hα-syn^+/–^ mice following rotenone treatment for 2 months. Furthermore, the number of activated microglia cells increased in SNc and accompanied the high expression of inflammatory cytokines, namely, TNF-α and IL-18 in the midbrain of old hα-syn^+/–^ mice treated with rotenone. Meanwhile, we found that after treatment with rotenone, hα-syn positive particles deposited in the intestinal wall, intestinal microflora, and T lymphocyte subtypes of Peyer’s patches changed, and intestinal mucosal permeability increased. Moreover, these phenomena were age-dependent. These findings suggested that rotenone aggravated the PD-like pathologies and affected the brain and gut of human α-syn^+/–^ transgenic mice in an age-dependent manner.

## Highlights

- Rotenone aggravated the PD-like pathology in the brain of hα-syn^+/–^ mice.
- Rotenone promoted PD-like pathology in the gut of hα-syn^+/–^ mice.
- Rotenone decreased the moving endurance of hα-syn^+/–^ mice with aging.
- Rotenone reduced dopaminergic neurons of hα-syn^+/–^ mice with aging.
- Rotenone induced early onset of gut pathology in the hα-syn^+/–^ mice.

## Introduction

Parkinson’s disease (PD) is the second most prevalent neurodegenerative disease worldwide, affecting more than 1% of the population over the age of 65. The typical pathology of PD is characterized by the formation of Lewy bodies (LBs) and the loss of dopaminergic neurons in the substantia nigra pars compacta (SNc) (Arotcarena et al., 2020). The major component of LBs is α-synuclein, in which fibrotic α-syn becomes the key to the LBs pathology due to its aggregation propensity (Spillantini et al., 1998). In addition to the central nervous system (CNS), the aggregates of α-syn fibrils were also found in the enteric nervous system (ENS) in patients with PD (Braak et al., 2006; Beach et al., 2016; Zhong et al., 2017; Manfredsson et al., 2018). New evidence strongly suggests that α-syn fibrils could spread from the ENS toward the brain and propagate across from one region to others of the brain (Kim et al., 2019; Challis et al., 2020; Gómez-Benito et al., 2020). There is an inevitable relationship between the production of intestinal α-syn and the intestinal microenvironment. Increasing evidence of brain–gut axis interaction is helpful to understand the pathological characteristics of the brain and gut in PD. The concept of the brain–gut axis has been further developed and enriched in the past decade, and accumulating evidence indicated that brain–gut interaction plays an important role in the pathological formation of neurodegenerative diseases (Arotcarena et al., 2020). Because of the complex influencing factors involved, the influence of the brain–gut axis on neurodegenerative diseases is still unclear (Kim et al., 2019).

Epidemiological studies have shown that environmental factors, such as pesticides, herbicides, and metals, increase the risk of developing PD (Ascherio and Schwarzschild, 2016; Bjorklund et al., 2018). The most commonly utilized neurotoxins to induce the animal models of PD are 6-hydroxydopamine, 1-methyl-4-phenyl-1, 2, 3, 6-tetrahydropyridine (MPTP), and rotenone (Simola et al., 2007; von Wrangel et al., 2015; Pupyshev et al., 2019). PD model triggered by rotenone, an inhibitor of mitochondrial complex I, may have some advantages over several other PD models (Cannon et al., 2009). Chronic administration of rotenone mimics behavioral changes and the key pathological feature of PD, including the intracellular α-synuclein aggregation (Betarbet et al., 2006; Alikatte et al., 2021). More recently, it has been found that chronic rotenone exposure can increase α-syn positive protein aggregates in the ENS (Drolet et al., 2009; Miyazaki et al., 2020). Dodiya et al. (2020) also found that rotenone alone reduced the tight junction protein expression, such as zonulae occludens protein 1 (ZO-1), and increased oxidative stress (Nitro-tyrosine) in the colon of mice. Studies have shown that 6-OHDA-induced PD mice have oxidative and/or nitrosation stress, and the expression of inducible nitric oxide synthase (iNOS) is increased and produces peroxynitrite and nitrotyrosine (Lee et al., 2011). Also, rotenone treatment enhanced astrocyte proliferation and α-syn expression in the colonic myenteric plexus of mice (Dodiya et al., 2020). These studies suggest that the administration of rotenone may provide a good means to investigate the interaction between the brain and gut in the development of PD.

Genetic susceptibility may determine the risk of PD in a particular individual, but it does not mean that it occurs definitely. Environmental factors may be the synergistic effect, accelerating the PD-like pathology in individuals with specific genotypes (George et al., 2010). One’s flexible adaptability declines with age, which accumulates pathological factors and eventually causes irreversible typical clinical symptoms. Whether environmental factors (rotenone exposure) act on the intestines, thereby accelerating the risk of α-syn mutation (hα-syn^+/–^) and leading to the formation of PD-like pathology in the brain and gut, still needs more systematic research. In particular, the cross-sectional studies of different ages are helpful to observe the key role of age in this process. Therefore, in this study, low-dose rotenone was administrated to 3-month-old and 9-month-old hα-syn^+/–^ (A53T) heterozygous transgenic mice for 2 months, and the changes in PD-like pathologies in the brain and gut were observed to explore the synergistic effects of environment–gene–age interaction in the induction of PD.

## Materials and methods

### Reagents

Rotenone (cat. 45656) and mouse monoclonal anti-α-synuclein antibody (cat. S5566) were purchased from Sigma Aldrich (Sigma, MO, United States). Mouse monoclonal anti-β-actin antibody (BM0627) and goat anti-mouse IgG antibody (BA1050) were purchased from Boster (Boster, CA, United States). Rabbit monoclonal to Iba-1 antibody (cat. ab178847) and rabbit polyclonal to tyrosine hydroxylase antibody (cat. ab41528) were purchased from Abcam (Abcam, Cambridge, United Kingdom). Anti-CD4 FITC (cat. 11-0041-81, GK1.5), anti-CD25 (cat. 45-0259-42, PerCPCyanine5.5), and anti-CD8a (cat. 12-0081-82, PE53-6.7) were purchased from Invitrogen (Invitrogen, CA, United States). Anti-mouse CD45 (cat. 564279, BUV395) was purchased from BD Horizon™ (BD, NJ, United States). RNA extraction kit, reverse transcription kit, and real-time PCR kit were purchased from Takara Biotechnology Co., Ltd. (Takara, Dalian, China).

### Animals

**FIGURE 1:**
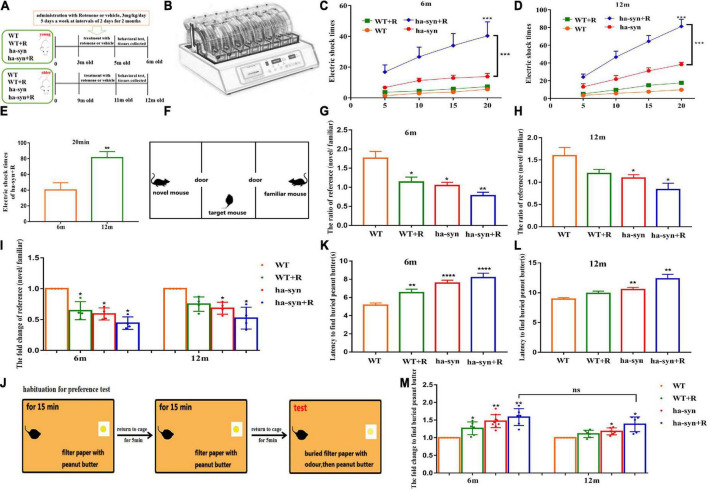
Rotenone significantly impaired the motor and non-motor function of hα-syn^+/–^ mice. **(A)** The experiment flowchart. Muscle endurance of mice was detected by wheeling test **(B–E)**. **(B)** Behavior paradigm of rotation wheel instrument. **(C)** The average number of electric shocks for 6-month-old hα-syn^+/–^ mice at 5, 10, 15, and 20 min. **(D)** The average number of electric shocks of 12-month-old hα-syn^+/–^ mice at 5, 10, 15, and 20 min. **(E)** The number of electric shocks of hα-syn + R mice during 20 min. Social recognition was detected using three-chamber equipment **(F–I)**. **(F)** The behavior paradigm of the three-chamber social test. **(G)** The effective contact time of 6-month-old hα-syn^+/–^ mice between the novel mice and familiar mice. **(H)** The effective contact time of 12-month-old hα-syn^+/–^ mice between the novel mice and familiar mice. **(I)** The fold change of contact time in mice. The olfactory function of mice was detected by exploring the peanut butter experiment **(J–M)**. **(J)** The behavior paradigm of olfaction test. **(K)** The time for the 6-month-old hα-syn^+/–^ mice to find the filter paper. **(L)** The time for the 12-month-old hα-syn^+/–^ mice to find the filter paper. **(M)** The fold change of contact time in mice. *n* = 6–9. *P < 0.05, **P < 0.01, ***P < 0.001, ****P < 0.0001, compared with the same age WT group. ns, there is no significance between groups.

Male human α-syn^+/–^ (A53T) transgenic mice (Giasson et al., 2002) (Jackson Laboratory, Stock No. 004479) were maintained by mating heterozygous transgenic mice with C57BL/6 mice (Jackson Laboratory, Stock No. 000664). The transgenic mice were identified *via* PCR analysis of total DNA by using specific primers which are shown in Table 1. The animals were bred and housed in the SPF laboratory of Lanzhou University at 20–25°C and 45–60% humidity and maintained under a 12 h light–dark cycle with food and water *ad libitum*. Mice were randomly divided into four groups: wild-type group (WT, C57BL/6 background), wild-type mice combined with rotenone group (WT + R), transgenic group (hα-syn^+/–^), and transgenic mice combined with rotenone group (hα-syn + R). In this experiment, 3 and 9 months wild-type or human α-syn^+/–^ (A53T) transgenic mice were used (Figure 1A). All animal experiments were approved by the Experimental Animal Ethics Committee of Lanzhou University.

**TABLE 1:** List of primer sequences.

Gene name	Forward (5′-3′)	Reverse (5′-3′)
*Transgenic (A53T)*	*TGTAGGCTCCAAAACCAAGG*	*TGTCAGGATCCACAGGCATA*
*Wild-type*	*CTAGGCCACAGAATTGAAAGATCT*	*GTAGGTGGAAATTCTAGCATCATCC*
*Bacteroides sp. (Bact)*	GGTTCTGAGAGGAGGTCCC	GCTGCCTCCCGTAGGAGT
*Lactobacillus sp. (Lact)*	*AGCAGTAGGGAATCTTCCA*	*CACCGCTACACATGGAG*
*Mouse Intestinal Bacteroides (MIB)*	*CCAGCAGCCGCGGTAATA*	*CGCATTCCGCATACTTCTC*
*Clostridium leptum (Clept)*	*GTTGACAAAACGGAGGAAGG*	*GACGGGCGGTGTGTACAA*
*Eubacterium rectale/(Erec)*	*ACTCCTACGGGAGGCAGC*	*GCTTCTTAGTCAGGTACCGTCAT*
*segmented filamentous bacteria (SFB)*	*GACGCTGAGGCATGAGAGCAT*	*GACGGCACGGATTGTTATTCA*
*total bacteria*	*ACTCCTACGGGAGGCAGCAGT*	*ATTACCGCGGCTGCTGGC*
*TNF*-α	*CCCTTTACTCTGACCCCTTTATTGT*	*TGTCCCAGCATCTTGTGTTTCT*
*IL-1*α	*TGGTTAAATGACCTGCAACAGGAA*	*AGGTCGGTCTCACTACCTGTGATG*
*IL-18*	*TTCTGCAACCTCCAGCATCA*	*AGTGAAGTCGGCCAAAGTTGTCT*
*GAPDH*	*GCGAGACCCCACTAACATCAA*	*GTGGTTCACACCCATCACAAA*

### Dosage regimen of rotenone

Rotenone was dissolved in 2% DMSO and 98% saline and preserved at 4°C (Pan-Montojo et al., 2010). WT mice and hα-syn^+/–^ mice (at the age of 3 and 9 months) were treated with 3 mg/kg/day rotenone solution (or vehicle control) for 5 consecutive days a week for 2 months.

### Behavior test

#### Wheeling test

To evaluate motor endurance, mice were subjected to the wheeling test using a rotation wheel instrument. Before the formal experiment, according to the early exploration of the physical conditions of mice, the instrument was set to medium difficulty with a parameter of 10 rpm, current 1.25 mA for 20 min. In the formal experiment, the number of electric shocks was recorded at the time points of 5, 10, 15, and 20 min, respectively.

#### Social recognition test

The device used in this test is a box (60 cm × 30 cm × 30 cm), which is divided averagely into three parts. The dividing walls were made of transparent plastic with a small square to allow the exploration of each chamber. In the habituation test, the target mouse was placed in the middle chamber to freely explore the entire apparatus for 10 min. In the sociability test, a novel male mouse that had no contact with the target mouse was placed in the left chamber, and a familiar male mouse that had contact with the target mice was placed in the right chamber, the target mice were placed in the middle chamber to freely explore the entire apparatus for 10 min again. The infrared camera system was used to record the effective contact time between the novel mice and familiar mice.

#### Olfaction test

The olfaction test was performed according to the previous methods (Cho et al., 2018). The experiment device is a clean mouse cage containing mouse padding (standard plastic cages, 25 cm × 15 cm × 12 cm). In the habituation training, a filter paper (5 cm × 5 cm) with peanut butter solution (0.6 g dissolved in 1 ml of distilled water) was placed on one side of this cage. After fasting for 24 h, the mice to be tested were put into the other side of this cage for 15 min and returned to the original cage for 5 min, and then the habituation training was repeated two times. In the formal experiment, a filter paper dripping with 100 μl of distilled water was buried under the padding, and the time taken for the mice to find the filter paper was recorded. After finding the filter paper, the mice were allowed to go back to the original cage and rest for 5 min. Again the filter paper dripping with 100 μl of peanut butter was buried under the padding, and the time taken for the mice to find the filter paper was recorded.

### Preparation of tissue

#### Preparation of brain sections

The mice were subjected to an intraperitoneal local anesthetic of 3% pentobarbital sodium (45 mg/kg, i.p.). Their thoracic cavity was opened, and 0.9% normal saline was perfused through the left ventricular and then replaced with 4% paraformaldehyde. The brain was fixed with 4% paraformaldehyde overnight and sank in 20 and 30% sucrose. Then, the mouse brain was embedded in the Tissue-Tek OCT compound (Sakura, Torrance, CA, United States) and frozen for sections. Serials of the coronal section were made using a freezing microtome at 25 μm, and brain sections were collected and placed in cryopreservation solution at −20°C for use.

#### Preparation of fresh brain tissue

The mice were anesthetized with phenobarbitone (45 mg/kg, i.p.). The midbrain was separated under the stereomicroscope, frozen with liquid nitrogen, and stored at −80°C before use.

#### Preparation of the small intestine tissue and Peyer’s patches

The mice were anesthetized with phenobarbitone (45 mg/kg, i.p.). Their abdominal cavity was opened, and the small intestine was fully exposed. Then, the small intestine from the duodenum to the cecum was aseptically removed, and the feces were flushed out with CMF/HEPES solution. When the Peyer’s patches were identified, they were excised and transferred into the iced CMF/HEPES. Then, the small intestine was cut and fixed with 4% paraformaldehyde overnight.

#### Preparation of the small intestine sections

The proximal small intestine was obtained and fixed in 4% PA solution overnight, then dehydrated with 30% sucrose, and the sagittal sections were made using a freezing microtome at a thickness of 15 μm.

### Immunofluorescence

According to the mouse brain atlas (Paxinos and Franklin, 2003), three sections were selected in the SNc: Bregma −2.7, Bregma −2.95, Bregma −3.2, interval 250 μm. The brain sections of SNc were selected and incubated with mouse monoclonal anti-α-synuclein antibody (1:200) at 37°C for 1 h and 4°C overnight. The sections were incubated with goat anti-mouse Cy3 antibody (Bioss, bs-0296G-Cy3, 1:200) at 37°C for 1 h. The sections were stained with DAPI and observed under the fluorescence microscope.

### β-Gal staining

The SNc sections were selected and fixed with 1% paraformaldehyde at room temperature for 20 min. The sections were rinsed and stained according to the β-gal kit protocol, and then counterstained with 1% neutral red for 3 min. Finally, the sections were dehydrated, sealed, and observed under the optical microscope.

### Immunohistochemistry

The small intestinal sections were quenched in 0.3% hydrogen peroxide (H_2_O_2_) for 20 min and then incubated with mouse monoclonal anti-α-synuclein antibody (1:200). The SNc sections were selected and quenched with 0.3% H_2_O_2_ for 20 min and then incubated with monoclonal to Iba-1 antibody (1:200) or polyclonal to tyrosine hydroxylase antibody (1:200) at 4°C overnight. The sections were incubated with biotinylated goat anti-rabbit IgG and horseradish enzyme-labeled streptomycin from Histostain™-Plus Kits (ZSGB-BIO, SP-9001) and immersed in 0.02% 3,3-diaminobenzidine containing 0.01% H_2_O_2_ in 0.01 M PBS for development. Finally, the sections were dehydrated, sealed, and observed under the optical microscope.

### Cell counts

At least three mice were selected from each group, and according to the mouse brain atlas (Paxinos and Franklin, 2003), three slices were selected from the substantia nigra brain region (Bregma −2.7, Bregma −2.95, and Bregma −3.2). After immunohistochemistry, the number of Iba-1^+^ or TH^+^ cells in SNc was counted separately by an observer blinded to the experiment.

### Intestinal permeability measurements

The intestinal permeability was measured as previously described (Yoseph et al., 2016). The mice were fasted and deprived of water for 4 h in advance before the intestinal permeability test to prevent confounding factors associated with dietary sugar intake. The mice were intragastrical administered with 4 kDa FITC-Dextran (FD4) at a concentration of 80 mg/ml. At 4 h later, the eyeball blood was collected, and the serum was separated and diluted by 0.01 M PBS. The fluorescence intensity of the serum was detected by a fluorescence spectrophotometer.

### Analysis of intestinal microbiota

The primers of intestinal microbiota were designed according to the literature (Wellman et al., 2017) and are shown in Table 1. The fecal total DNA was extracted by TIANamp Stool DNA Kit (TIANGEN Biotech, Co., Ltd., Beijing, China). The DNA levels of some intestinal microbiota in triplicate samples were evaluated with an SYBR Green PCR Master Kit (Promega Corporation, United States) following the manufacturer’s instructions. The assays were initiated for 5 min at 95°C, 40 cycles of 15 s at 94°C, and 1 min at 60°C. The amplification of target microbiota DNA was normalized to the expression of total bacteria. The relative DNA expression levels of target microbiota were calculated by using a 2^–ΔΔCT^ method.

### Western blot

For protein expression analysis of α-syn, the protein was extracted from the midbrain with RIPA buffer containing protease inhibitors, phosphatase inhibitors, and phenylmethylsulfonyl fluoride (Beyotime, Shanghai, China). The tissue was then mechanically homogenized, followed by incubation on ice for 2 h, sonication for 3 min, and centrifuged at 12,000 rpm at 4°C for 30 min to take the supernatant and quantify.

For each sample, 100 μg of protein was loaded into 10% gels without denaturalization, and electrophoresis was performed at 60 V for 1 h to concentrate and 110 V for 1 h to separate at 4°C. Then, the proteins were transferred to PVDF membranes at 250 mA for 1 h at 4°C. Membranes were blocked with 5% skim milk for 1 h, washed with TBST (containing 0.1% Tween-20), and incubated at 4°C with anti-α-synuclein antibody (1:2,000) and anti-β-actin antibody (1:3,000) overnight. The next day, the membranes were washed and incubated with goat anti-mouse IgG antibody (1:500) for 1 h at RT, rewashed, and exposed with ECL (Millipore, Bedford, MA, United States). The bands were visualized using the Azure (c500) imaging system (Azure, CA, United States), and the Western blot results were analyzed by ImageJ software.

### Q-RT-PCR

Total RNA was extracted from the midbrain using an RNAiso Plus reagent (Takara Biotech, Co., Ltd., Dalian, China) under the manufacturer’s instructions. One microgram of total RNA was used for cDNA synthesis with M-MuLV reverse transcriptase according to the manufacturer’s instructions. Target mRNAs were detected and quantified by a PIKoREAl96 detector (Thermo Scientific, United States). The nucleotide sequences of primers are shown in Table 1. SYBR Green PCR Master Kit (Promega Corporation, United States) was used with the following PCR parameters, 5 min at 95°C, 40 cycles of 15 s at 94°C, and 1 min at 60°C. The results were calculated by using a 2^–ΔΔCT^ method normalized against the housekeeping gene GAPDH.

### Flow cytometry

Peyer’s patches were isolated from the mouse’s small intestine and then gently forced the tissue through a nylon grid with forceps. The obtained cell suspension was precipitated by gravity at 4°C CMF/HEPES for 10 min, the plastic particles were discarded, and the supernatant was collected. The cells in the supernatant were washed in CMF/HEPES three times. The cells were resuspended in 0.01 M PBS and the cell concentration was adjusted to 10^5^–10^6^ cells/ml. Then, the cells were washed with 0.01 M PBS, and 0.5 μl of anti-CD4, 1.25 μl of anti-CD8, 2 μl of anti-CD45, and 1.25 μl of anti-CD25 were added and incubated at 4°C for 30 min in the dark. After washing the cells, 300–500 μl of flow buffer was used to resuspend the cells. Flow cytometry was used to detect the lymphocyte subsets and calculate the changes in different lymphocyte subsets.

### Statistical analysis

The immunohistochemical image statistics were quantified by Image-Pro Plus, and the immunofluorescence and Western blot results were analyzed by the ImageJ software, respectively. All data were expressed as mean ± SEM. All statistical analyses were carried out using SPSS17.0 statistical software. The multiple group comparisons were analyzed by one-way or two-way ANOVA. The significance between the two groups was evaluated by the *t*-test analysis. A *P*-value of <0.05 was considered significant.

## Results

### Effect of rotenone on motor and non-motor functions in hα-syn^+/–^ mice

In the present study, the motor coordination and endurance of mice were evaluated by the spinning wheel equipment. The results showed that the number of electric shocks increased with longer testing time in 3-month or 9-month-old hα-syn^+/–^ mice treated with rotenone for 2 months, suggesting a little effect on motor coordination but a significant decrease in motor endurance (Figures 1B–D). The number of electric shocks increased 2-fold in the 12-month-old hα-syn^+/–^ mice compared with the 6-month-old hα-syn^+/–^ mice of the hα-syn + R group (Figure 1E), suggesting that rotenone causes a more pronounced decrease in motor endurance with increasing age in hα-syn^+/–^ mice. PD is often accompanied by a decrease in cognitive function, and we tested the social recognition ability of mice with three-chamber equipment. The decline of social recognition was observed in 6-month and 12-month-old hα-syn^+/–^ mice with intragastric administration of rotenone for 2 months (Figures 1F–H) and decreased to 50% in 6-month and 12-month-old hα-syn^+/–^ mice compared to the controls of the same age (Figure 1I), suggesting that the decrease in social recognition is already significant in 6-month-old hα-syn^+/–^ mice. Olfactory hypofunction is also a common complication of PD, and the odor recognition experiments showed longer time spent in 6-month and 12-month-old hα-syn^+/–^ mice with rotenone compared with the controls (Figures 1J–L). However, there was no significant difference in the exploration time between 6-month and 12-month-old hα-syn^+/–^ mice with rotenone compared with the age-matched controls (Figure 1M), which suggests that the effect of rotenone on olfactory sensitivity in hα-syn^+/–^ mice is more pronounced at an early stage.

### Effect of rotenone on the survival of DA neurons in the SNc of hα-syn^+/–^ mice

**FIGURE 2:**
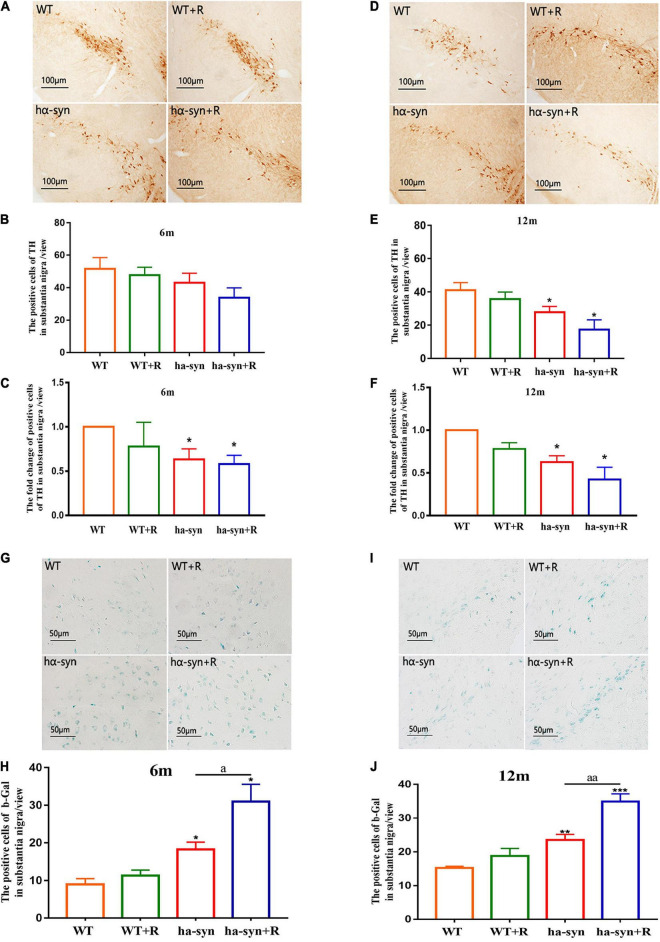
Rotenone significantly decreased the number of TH-positive neurons and promoted cell senescence in the SNc of hα-syn^+/–^ mice. The immunohistochemical technique combined with cell counting was used to detect the number of TH-positive neurons in the SNc of mice of different ages **(A–F)**. **(A)** Representative images showing TH-positive neurons in SNc of 6-month-old hα-syn^+/–^ mice. **(B)** The number of TH-positive neurons in SNc of 6-month-old hα-syn^+/–^ mice. **(C)** The fold change of TH-positive neurons in SNc of 6-month-old hα-syn^+/–^ mice compared with the WT group. **(D)** Representative images showing TH-positive neurons in SNc of 12-month-old hα-syn^+/–^ mice. **(E)** The number of TH-positive neurons in SNc of 12-month-old hα-syn^+/–^ mice. **(F)** The fold change of TH-positive neurons in SNc of 12-month-old hα-syn^+/–^ mice compared with the WT group. The senescent cells in the SNc of different ages were detected by β-gal staining combined with cell counting **(G–J)**. **(G)** Representative images showing β-gal staining in SNc of 6-month-old hα-syn^+/–^ mice. **(H)** A number of positive cells of β-gal in SNc of 6-month-old hα-syn^+/–^ mice. **(I)** Representative images showing β-gal staining in SNc of 12-month-old hα-syn^+/–^ mice. **(J)** A number of positive cells of β-gal in SNc of 12-month-old hα-syn^+/–^ mice. *n* = 6. *P < 0.05, **P < 0.01, ***P < 0.001, compared with the age-matched WT group. *^a^P* < 0.05, *^aa^P* < 0.01, compared with the age-matched hα-syn^+/–^ group.

The results showed that the number of DA neurons in the SNc of the midbrain was significantly reduced in 3-month and 9-month-old hα-syn^+/–^ mice with rotenone for 2 months compared with the controls (Figures 2A–F). The reduction was about 40% in 6-month-old hα-syn^+/–^ mice compared with the age-matched controls (Figure 2C) and about 60% in 12-month-old hα-syn^+/–^ mice compared with the age-matched controls (Figure 2F), suggesting that the loss of DA neuron in the SNc worsened with age. Considering the risk of aging, we examined the number of aging cells in the SNc based on β-gal staining, and the results showed a significant increase in the number of β-gal-positive cells in the SNc of rotenone-treated hα-syn^+/–^ mice (Figures 2G–J).

### Effect of rotenone on hα-syn expression and microglia activation in the midbrain of hα-syn^+/–^ mice

**FIGURE 3:**
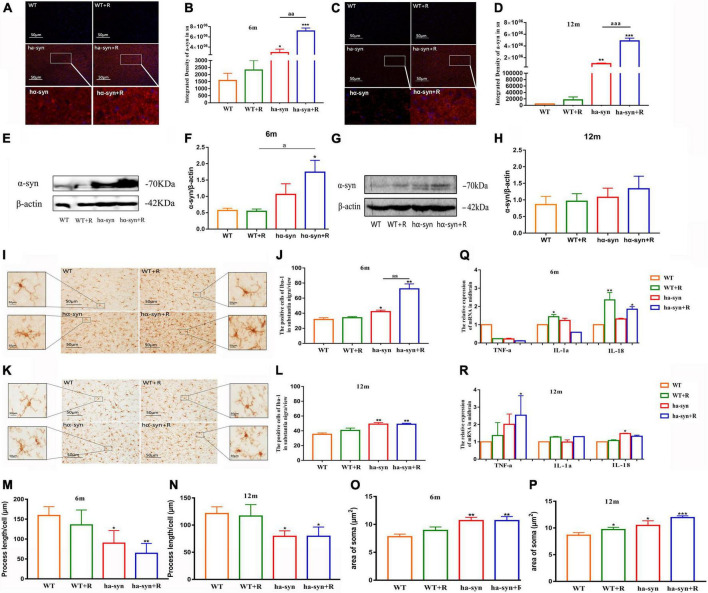
Rotenone increased the expression of α-syn and promoted the proliferation and heterogeneity of microglia in the SNc of hα-syn^+/–^ mice. Semiquantitative analysis of α-syn expression in SNc by immunofluorescence technique **(A–D)**. **(A)** Representative images of α-syn expression in the SNc of 6-month-old hα-syn^+/–^ mice, α-syn (red), DAPI (blue). **(B)** Fluorescence intensity analysis of α-syn expression in the SNc of 6-month-old hα-syn^+/–^ mice by the ImageJ software. **(C)** Representative images of α-syn expression in the SNc of 12-month-old hα-syn^+/–^ mice, α-syn (red), DAPI (blue). **(D)** Fluorescence intensity analysis of α-syn expression in the SNc of 12-month-old hα-syn^+/–^ mice by ImageJ software. Semiquantitative analysis of α-syn expression in the midbrain by Western blot **(E–H)**. **(E)** Representative WB images of α-syn expression in the midbrain of 6-month-old hα-syn^+/–^ mice. **(F)** The WB images of α-syn expression in the midbrain of 6-month-old hα-syn^+/–^ mice were scanned and computed the gray score. **(G)** Representative WB images of α-syn expression in the midbrain of 12-month-old hα-syn^+/–^ mice. **(H)** The WB images of α-syn expression in the midbrain of 12-month-old hα-syn^+/–^ mice were scanned and computed the gray score. The number of microglia in SNc of mice of different ages was detected by immunohistochemical technique combined with cell counting **(I–L)**. **(I)** Representative images showing Iba-1-positive cells in SNc of 6-month-old hα-syn^+/–^ mice. **(J)** A number of Iba-1-positive cells in SNc of 6-month-old hα-syn^+/–^ mice. **(K)** Representative images showing Iba-1-positive cells in SNc of 12-month-old hα-syn^+/–^ mice. **(L)** A number of Iba-1-positive cells in SNc of 12-month-old hα-syn^+/–^ mice. Morphological analysis of microglia by Image J **(M-P)**. **(M)**, Statistical results of process length of microglia at the age of 6 months. **(N)**, Statistical results of process length of microglia at the age of 12 months. **(O)**, Statistical results of the area of microglial soma at 6 months old. **(P)**, Statistical results of the area of microglial soma at 12 months old. Analysis of levels of inflammatory cytokines in the midbrain of mice of different ages by qPCR **(Q-R)**. **(Q)**, mRNA level of TNF-α, IL-1α, IL-18 in the midbrain of 6-month-old mice. **(R)**, mRNA level of TNF-α, IL-1α, IL-18 in the midbrain of 12-month-old mice. *n* = 6. *P < 0.05, **P < 0.01, ***P < 0.001, compared with the age-matched WT group. *^a^P* < 0.05, compared with the age-matched WT + R group. *^aa^P* < 0.01, *^aaa^P* < 0.001, compared with the age-matched hα-syn^+/–^ group.

Using immunofluorescence histochemistry combined with semi-quantitative analysis of fluorescence intensity, it was found that rotenone significantly increased α-syn levels in the SNc of hα-syn^+/–^ mice at the age of 6 and 12 months compared with the age-matched controls (Figures 3A–D). The results of WB are consistent with those of immunofluorescence quantification (Figures 3E–H). Considering the inflammatory characteristics of α-syn, we analyzed the number and morphology of microglia in the SNc, and the results showed that rotenone promoted the proliferation and phenotypic transformation of microglia in the SNc of hα-syn^+/–^ mice. The number of activated microglia were significantly increased compared with the control group (Figures 3I–L), and the process of microglia became shorter (Figures 3M,N) as the area of microglia soma became larger (Figures 3O,P). Furthermore, we examined the levels of inflammatory factors in the midbrain and found that in 6-month-old hα-syn^+/–^ mice, rotenone mainly increased the expression of IL-18 (Figure 3Q), which may indicate that activated microglia are more associated with inflammasome signaling. In 12-month-old hα-syn^+/–^ mice, rotenone mainly increased the expression of TNF-α (Figure 3R). The above results suggest that there is heterogeneity in microglial activation in the midbrain of hα-syn^+/–^ mice caused by rotenone.

### Effect of rotenone on the intestinal α-syn expression and intestinal permeability in hα-syn^+/–^ mice

**FIGURE 4:**
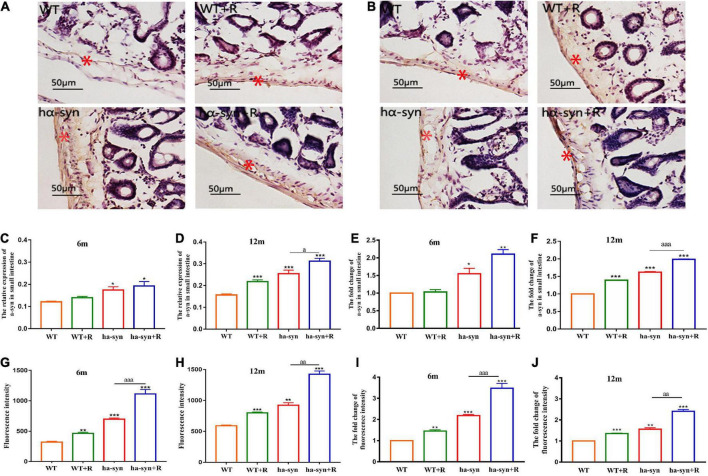
Rotenone significantly elevated intestinal α-syn levels in hα-syn^+/–^ mice and may affect intestinal permeability. The expression levels of α-syn in the intestinal were detected by immunohistochemical technique at different ages **(A–F)**. **(A)** Representative images of α-syn expression in the small intestine of 6-month-old hα-syn^+/–^ mice, and the red asterisk represents the positive area. **(B)** Representative images of α-syn expression in the small intestine of 12-month-old hα-syn^+/–^ mice, and the red asterisk represents the positive area. **(C)** Grayscale analysis of α-syn expression in the small intestine of 6-month-old hα-syn^+/–^ mice by ImageJ. **(D)** Grayscale analysis of α-syn expression in the small intestine of 12-month-old hα-syn^+/–^ mice by the ImageJ software. **(E)** The fold change of α-syn expression in the small intestine of 6-month-old hα-syn^+/–^ mice compared with the WT group. **(F)** The fold change of α-syn expression in the small intestine of 12-month-old hα-syn^+/–^ mice compared with the WT group. The FITC fluorescence is used to evaluate intestinal mucosal permeability **(G–J)**. **(G)** Fluorescence intensity analysis of FITC in the serum of 6-month-old hα-syn^+/–^ mice. **(H)** Fluorescence intensity analysis of FITC in the serum of 12-month-old hα-syn^+/–^ mice. **(I)** The fold change of FITC in the serum of 6-month-old hα-syn^+/–^ mice compared with the WT group. **(J)** The fold change of FITC in the serum of 12-month-old hα-syn^+/–^ mice compared with the WT group. *n* = 6. *P < 0.05, **P < 0.01, ***P < 0.001, compared with the age-matched WT group. *^a^P* < 0.05, *^aa^P* < 0.01, *^aaa^P* < 0.001, compared with the age-matched hα-syn^+/–^ group.

The results showed that rotenone significantly increased the level of intestinal α-syn in 6-month and 12-month-old hα-syn^+/–^ mice compared with the controls (Figures 4A–D). Among them, in 6-month-old hα-syn^+/–^ mice, there was a 2.3-fold increase compared with the age-matched control group (Figure 4E), and in 12-month hα-syn^+/–^ mice, there was about a 2-fold increase compared with the age-matched control group (Figure 4F). Furthermore, the intestinal mucosal permeability was evaluated using Dextran 40-FITC gavage and measured the level of FITC fluorescence in the blood after 4 h. The results showed that rotenone significantly increased FITC fluorescence intensity in the serum of hα-syn^+/–^ mice compared with the controls (Figures 4G,H). In 6-month-old hα-syn^+/–^ mice, there is a 3-fold increase compared to the age-matched controls (Figure 4I) and in 12-month-old hα-syn^+/–^ mice, there is a 2.5-fold increase compared to age-matched controls (Figure 4J). These results suggested that rotenone increased the level of intestinal α-syn in hα-syn^+/–^ mice in early life and may affect intestinal permeability.

### Effect of rotenone on the intestinal flora of hα-syn^+/–^ mice

**FIGURE 5:**
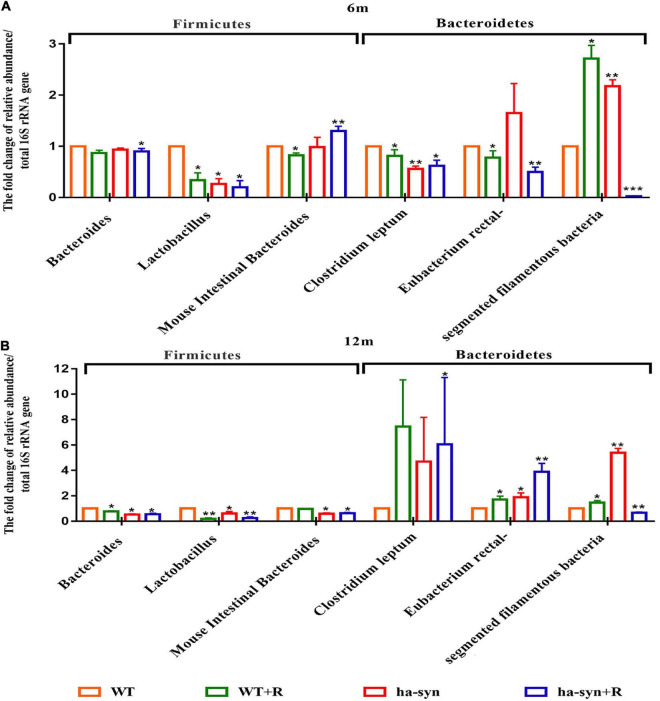
The effect of rotenone on the intestinal bacteria of hα-syn^+/–^ mice. The changes of specific intestinal bacteria in the mice feces of different ages were detected by qPCR. **(A)** Changes of specific beneficial and harmful bacteria in the small intestine of 6-month-old hα-syn^+/–^ mice. **(B)** Changes of specific beneficial and harmful bacteria in the small intestine of 12-month-old hα-syn^+/–^ mice. *n* = 6. *P < 0.05, **P < 0.01, ***P < 0.001, compared with the age-matched WT group.

We selected beneficial and harmful bacteria closely related to PD and examined the effect of rotenone on their growth abundance. The results showed that the abundance of the beneficial bacteria such as *Lact* reduced and the harmful bacteria such as *SFB* increased in 6-month-old hα-syn^+/–^ mice compared with the controls (Figure 5A). In 12-month-old hα-syn^+/–^ mice, the proportion of the beneficial bacteria such as *Lact and Bact* significantly reduced, while the harmful bacteria such as *Erec* increased compared with the controls (Figure 5B). These results suggested that rotenone has influenced the stability of the intestinal flora in hα-syn^+/–^ mice.

### Effect of rotenone on subpopulations of the lymphocytes in Peyer’s patches in hα-syn^+/–^ mice

**FIGURE 6:**
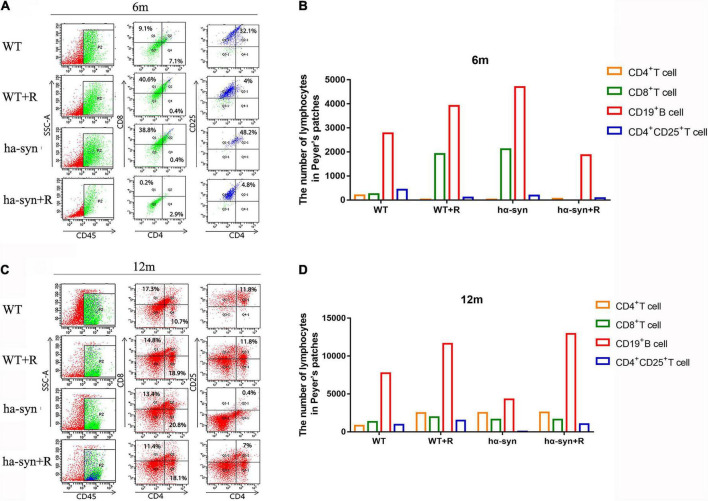
The effect of rotenone on subpopulations of Peyer’s patches lymphocytes in hα-syn^+/–^ mice. Flow cytometry was used to detect the changes in lymphocyte subsets, namely, CD4^+^, CD8^+^, and CD4^+^CD25^+^ lymphocytes, respectively, in Peyer’s patches in mice of different ages. **(A)** Changes of lymphocyte subsets in Peyer’s patches of 6-month-old hα-syn^+/–^ mice by flow cytometry, *n* = 6. **(B)** Statistics of the number of cells in Peyer’s patches of 6-month-old hα-syn^+/–^ mice. **(C)** Changes of lymphocyte subsets in Peyer’s patches of 12-month-old hα-syn^+/–^ mice by flow cytometry, *n* = 6. **(D)** Statistics of the number of cells in Peyer’s patches of 12-month-old hα-syn^+/–^ mice.

Given the altered intestinal flora and the response of intestinal immune cells are closely related, we analyzed the changes in the subpopulation of Peyer’s patches lymphocytes at the intestinal site using flow cytometry. The results showed that the cell number of CD4^+^, CD8^+^, and CD4^+^CD25^+^ lymphocytes significantly reduced, respectively, in 6-month-old hα-syn^+/–^ mice compared with age-matched controls (Figures 6A,B). In the 12-month-old hα-syn^+/–^ mice, the cell number of CD8^+^ and CD4^+^CD25^+^ lymphocytes reduced and CD4^+^ lymphocytes increased compared with age-matched controls (Figures 6C,D).

## Discussion

Parkinson’s disease is an age-dependent progressive neurodegenerative disease. Genetic mutations, environmental toxicity, and increasing age interact to promote the progression of PD pathology. Pathological changes of PD in the brain are characterized by the loss of dopaminergic neurons in nigrostriatal and the formation of Lewy bodies consisting of alpha-syn mainly. The changes in peripheral organs such as the gastrointestinal nervous system, typically the change of intestinal alpha-syn aggregation in the intestinal flora. The main symptoms are characterized by a decrease in motor function, such as tremors and unsteady walking, and altered gastrointestinal function, such as abnormal bowel movements and impaired gastrointestinal absorption. However, it is not clear whether the temporal relationship and factors influence the development of peripheral and central pathology. In this study, we used hα-syn^+/–^ mice with low-dose rotenone gavage and observed the pathological characteristics in 6-month and 12-month-old hα-syn^+/–^ mice. The results revealed that rotenone aggravated motor dysfunction and DA neuron loss in the SNc of hα-syn^+/–^ mice in the age-dependence, with more pronounced changes in 12-month-old hα-syn^+/–^ mice. The effects of rotenone on cognitive function and gastrointestinal function in hα-syn^+/–^ mice showed early onset characteristics. The effects of rotenone on intestinal flora and lymphocyte subpopulation of Peyer’s patches in hα-syn^+/–^ mice reflected different characteristics at the age of 6 and 12 months.

Alpha-synuclein is the first protein found to be associated with familial hereditary PD (Chartier-Harlin et al., 2004). So far, the mutation sites of the SNCA gene encoding human α-syn protein include A30P, E46K, H50Q, G51D, A53E, A29S, and A53T, among which A53T has been deeply studied (Chartier-Harlin et al., 2004; de Oliveira and Silva, 2019). The work of several research groups has shown that human α-syn with A53T mutation is more likely to form fibrils, and fibrotic α-syn is prone to form aggregates, which is the main component of LBs (Li et al., 2004; Wan and Chung, 2012). Other studies have also shown that α-syn aggregates may be the main form transferred from the intestine to the brain (Challis et al., 2020), as well as the main form spread across brain regions (Niu et al., 2018; Gómez-Benito et al., 2020). Our studies showed that the level of α-syn in SNc of hα-syn^+/–^ (A53T) mice elevated significantly, and rotenone treatment had a synergistic effect.

Rotenone, as an insecticide and herbicide, is a lipophilic mitochondrial toxin that can cross the blood–brain barrier (Radad et al., 2019). It increases oxidative stress and inhibits the production of ATP by inhibiting the mitochondrial complex I activity, resulting in the insufficient energy supply of cells, mitochondrial dysfunction, neuronal damage, and intestinal pathology in the pathogenesis of PD (Tanner et al., 2011), and rotenone can promote the formation of α-syn and its inclusion body by free radical pathway (Betarbet et al., 2000). After long-term intragastric administration of low-dose rotenone, α-syn aggregation was observed in the ENS, the dorsal vagus nucleus, the medial-lateral nucleus of the spinal cord, brainstem, and SNc. The ENS and dorsal vagus nucleus also appeared inflammation and α-syn phosphorylation (Pan-Montojo et al., 2010). Several studies have found that rotenone can act on primary enteric neurons to promote α-syn secretion (Pan-Montojo et al., 2012). The secreted α-syn is absorbed by the presynaptic neurons of the sympathetic nerve, and then reversely transported to the cell body and aggregated in the brain (Chandra et al., 2005; Burré et al., 2010). Other studies have shown that rotenone may impede the energy-dependent ubiquitin–proteasome pathway of protein degradation, thereby increasing the α-syn aggregation (Johnson and Bobrovskaya, 2015). Our study found that hα-syn^+/–^ (A53T) mice combined with rotenone resulted in motor dysfunction in an age-dependent manner. Meanwhile, the number of dopaminergic neurons in the SNc decreased significantly at the age of 12 months.

More and more literature showed that the interaction of the brain–gut–microbiota axis may be involved in the pathogenesis of PD (Forsyth et al., 2011; Mulak and Bonaz, 2015; Arotcarena et al., 2020; Dodiya et al., 2020). We found that hα-syn^+/–^ mice combined with rotenone treatment increased the level of α-syn in the intestinal, and increased intestinal permeability, indicating that intestinal function was affected. The intestinal dysfunction may cause by the alteration of gut microbiota. Recent evidence from several laboratories (Keshavarzian et al., 2015; Scheperjans et al., 2015; Bedarf et al., 2017; Hill-Burns et al., 2017) proposes a relationship between the complexity and diversity of the microorganisms that inhabit our gut and PD progression. Indeed, even an appendectomy was recently seen as a potential prophylactic for PD initiation (Killinger et al., 2018). Studies have shown that the abundance of *Prevotellaceae* in feces of patients with PD was reduced compared with controls (Scheperjans et al., 2015). Another study also found that the abundance of *Lachnospiraceae, Erysipelotrichaceae, Prevotellaceae, Clostridiales, Erysipelotrichales*, and *proteobacteria* of the gut microbiota was changed significantly in an MPTP mouse model of PD (Lai et al., 2018). Some literature has shown that intestinal flora can regulate the activation of microglia in the PD model by producing short-chain fatty acids (Erny et al., 2015; Sampson et al., 2016). We further studied the effect of this change in the intestinal microbiota and found that the number of beneficial bacteria was significantly reduced and the number of harmful bacteria was significantly increased in the hα-syn^+/–^ mice treated with rotenone. Campos-Acuña et al. suggested that some components of intestinal microbiota might trigger the production of α-synuclein inclusions in the intestine, which is the main source of autoantigens that drive the immune response in PD. Moreover, gut microbiota produces a variety of mediators in the gut mucosa, such as SCFAs, dopamine, and other metabolites, which stimulate their receptors in the T cells, thus shaping the adaptive immune response (Campos-Acuña et al., 2019). Furthermore, Chen et al. (2015) found that patients with PD with constipation are associated with immune activation in the colonic mucosa. So, the T lymphocyte subsets of intestinal Peyer’s patches were analyzed by flow cytometry and found that the T lymphocyte subsets of the intestinal Peyer’s patches were also altered. The changes in the lymphocyte subsets of the intestinal are capable of driving intestinal inflammation, which not only causes pathological α-syn to spread to the brain but also affects the brain itself (Sampson et al., 2016).

Our study also found that the activation of microglia in the midbrain was significantly increased in the hα-syn^+/–^ mice treated with rotenone, and the expression of TNF-α in the midbrain was increased significantly in the 12-month-old hα-syn^+/–^ mice combined with rotenone treatment group, indicating that the microglia tend to its activated phenotype. In addition, we also detected the expression of the inflammasome in the midbrain. The mRNA level of IL-18 was upregulated after rotenone treatment, but there was no significant difference in the expression of NLRP3 (the data are not shown in this paper). Our results showed that after 2 months of intragastric administration of rotenone, the proportion of intestinal immune cell subsets and the level of intestinal segmental filamentous bacteria decreased in 6-month-old hα-syn^+/–^ mice. At the same time, microglia in substantia nigra were activated in 6-month-old hα-syn^+/–^ mice. These results suggested that the inflammatory immune mechanism may be involved in the pathogenesis of PD through the gut–brain axis.

Although we did not track the level of rotenone across the blood–brain barrier after peripheral administration in this study, our experimental results suggested that the gavage of rotenone alone was able to induce the loss of TH-positive neurons and microglia activation in the SNc of mice. Similarly, studies from other laboratories suggested rats treated with rotenone by gavage exhibited the pathological features of PD (Betarbet et al., 2000; Cannon et al., 2009; Miyazaki et al., 2020). The evidence indicates that rotenone can induce dopamine neuron loss across the blood–brain barrier, and also alters the intestinal microenvironment, which may result from the effects of rotenone on the intestinal flora and the mucosal barriers of the gastrointestinal tract. Our results suggested that the administration of rotenone alone also affected the abundance of intestinal flora and the permeability of the small intestinal mucosa. Our results showed that after 2 months of intragastric administration of rotenone in 3-month-old hα-syn^+/–^ mice, the level of α-syn in the small intestine increased by 50% compared with hα-syn^+/–^ mice of the same age. After 2 months of intragastric administration of rotenone in 9-month-old hα-syn^+/–^ mice, the level of α-syn in the small intestine was consistent with that in 6-month-old hα-syn^+/–^ mice with intragastric administration of rotenone for 2 months, which indicated that the effect of rotenone on intestinal pathology occurs in 3-month-old hα-syn^+/–^ mice. However, the loss of TH-positive neurons in substantia nigra and the decrease of motor ability were more obvious in 9-month-old hα-syn^+/–^ mice with intragastric administration of rotenone for 2 months, indicating that the neuronal damage effect of rotenone appears following the intestinal changes. These results suggested that environmental factors aggravate the incidence of PD in individuals with genetic susceptibility in the early stage, which is also consistent with the sequence of clinical symptoms in patients with PD, that is, gastrointestinal symptoms often appear in the early stage, followed by changes in motor symptoms (Knudsen et al., 2017).

Our study also has limitations, first, we did not clarify the possible pathways by which rotenone exacerbated α-syn expression in the small intestine. Second, the changes in intestinal flora and subpopulations of immune cells in the small intestine are only preliminary studies, and more experiments are needed to demonstrate how the interactions of the intestinal microenvironment and genetic susceptibility exacerbate the development of PD.

In summary, our present study showed that rotenone and α-syn interaction aggravated the PD-like pathology, in an age-dependent manner, by the brain-gut axis. In the future, it remains to be evaluated whether improving the gut microenvironment ameliorated the brain pathology of PD.

## Data availability statement

The original contributions presented in the study are included in the article/supplementary material, further inquiries can be directed to the corresponding author/s.

## Ethics statement

The animal study was reviewed and approved by Ethics Committee of Basic Medical School of Lanzhou University.

## Author contributions

A-DC and J-XC processed the experimental data, performed the analysis, drafted the manuscript, and designed the figures. H-CC, H-LD, X-XX, JS, and JY were involved in planning and supervised the work. Y-HJ and L-PG were aided in interpreting the results and worked on the manuscript. All authors discussed the results and commented on the manuscript.
